# Ghrelin protects against cell death in myocardial ischaemia: Emerging role of microRNA

**DOI:** 10.1113/EP093314

**Published:** 2026-01-29

**Authors:** Rajesh Katare, Jeffrey R. Erickson, Daryl O. Schwenke

**Affiliations:** ^1^ Department of Physiology, School of Biomedical Sciences University of Otago Dunedin New Zealand

**Keywords:** apoptosis, ghrelin, microRNA, myocardial infarction

## Abstract

Acute myocardial infarction (MI) accelerates cardiomyocyte apoptosis, which underpins ventricular remodelling and dysfunction. The hormone ghrelin mitigates this remodelling, but the mechanisms remain unclear. Specific microRNAs (miRs) are key modulators and reliable biomarkers of early‐stage apoptosis. We hypothesized that ghrelin targets anti‐apoptotic miR‐499 and miR‐133 following MI to suppress cardiac apoptosis and thus mitigate cardiac dysfunction. C57/B6 mice received an injection of ghrelin (150 µg/kg, s.c.) or saline following left anterior descending coronary artery ligation (MI). Plasma levels of miR‐499 and miR‐133 at 3 or 24 h post‐MI were measured using real‐time PCR. Echocardiography and TUNEL staining were used to assess progressive cardiac function/structure and cardiomyocyte apoptosis, respectively. Myocardial ischaemia adversely decreased the levels of anti‐apoptotic miR‐499 by 3 h post‐MI and increased the proportion of TUNEL‐positive apoptotic cardiomyocytes by 24 h post‐MI, contributing to cardiac remodelling and dysfunction by 2 weeks post‐MI. Ghrelin prevented this MI‐induced decrease in miR‐499 by 3 h post‐MI, then further increased the levels of miR‐499 and miR‐133 by 24 h. These ghrelin‐mediated changes in microRNA were associated with a significant decrease in cardiomyocyte apoptosis and, consequently, significantly improved cardiac function and structure by 2 weeks post‐MI. These results highlight miRs as effective biomarkers for the early detection of ischaemia‐induced apoptotic signalling. Moreover, ghrelin appears to mitigate ischaemia‐induced apoptosis by increasing the levels of anti‐apoptotic miR‐499 and miR‐133, further solidifying ghrelin as a new therapeutic strategy for the clinical treatment of heart failure.

## Abstract (highlights)

- **What is the central question of this study?**
  An acute myocardial infarction is associated with accelerated cardiomyocyte apoptosis. Ghrelin attenuates cardiomyocyte apoptosis, although the cellular pathways remain unclear. A potential role of microRNA in mediating the anti‐apoptotic effects of ghrelin has not been explored previously.
- **What is the main finding and its importance?**
  We show that acute myocardial infarction is associated with dysregulation of apoptotis‐specific microRNAs and that ghrelin treatment mitigates these changes in microRNA, thereby reducing cardiomyocyte apoptosis.

## 1 INTRODUCTION

Ever since its discovery in 1999 (Kojima et al., 1999), the peptide hormone ghrelin (28 amino acids), which is released from the stomach wall, has emerged as an important therapeutic modulator of cardiac function in health and disease (Kojima & Kangawa, 2005). Ghrelin has been shown to: (1) inhibit sympathetic nerve activity following myocardial infarction (Schwenke et al., 2008); (2) improve blood flow in ischaemic conditions by direct vasodilatation, reducing inflammation and promoting angiogenesis (Neale et al., 2020); and (3) reduce cardiac dysfunction in chronic heart failure (Schwenke et al., 2012). Emerging evidence suggests that ghrelin also inhibits programmed cell death (apoptosis) (Zhang et al., 2013), suggesting a potential clinical role for ghrelin in the prevention of cell death and fibrotic remodelling following acute myocardial infarction (MI).

Apoptosis is a tightly controlled cellular process that ensures the timely self‐termination of cells. In the heart, accelerated cardiomyocyte apoptosis plays a major role in the detrimental cardiac structural changes following an MI. Enhanced apoptotic signalling also reduces the population of viable depolarizing myocytes, which are replaced with electrically inert fibrotic tissue. This process contributes to the development of ischaemia‐induced arrhythmia following an acute MI and is thought to play a key role in mortality (van Heerebeek et al., 2008).

Conventional methods of measuring apoptosis have advanced our understanding of key mechanisms underpinning apoptotic signalling. Typically, such methods necessitate the collection of tissue samples/biopsies, and the sensitivity for detection is most apparent 12–24 h post‐MI. Apoptotic signalling is preceded by enhanced production of microRNAs (miRs) within a few hours following myocardial ischaemia (D'Alessandra et al., 2010; Pan et al., 2012). MicroRNAs are short non‐coding RNAs that modulate gene expression by targeting mRNA post‐transcription and, moreover, have pleiotropic effects on various target proteins (Rawal et al., 2014b). In general, individual miRs are tissue specific but are released into the circulation. Advantageously, this means that plasma levels of miRs collected from a simple blood sample provide important insight into the accelerated activation of apoptotic signalling in the early stages following myocardial ischaemia (Rawal et al., 2014b).

We have previously demonstrated in an experimental model of MI that immediate treatment with ghrelin (within 30 min post‐MI) significantly attenuates the magnitude of cardiac dysfunction and remodelling in chronic heart failure (Schwenke et al., 2012, 2008). However, the cardioprotective effects of ghrelin are greatly diminished when treatment is delayed, for example, 24 h post‐MI. Although the cellular mechanisms that underlie the protective effects of ghrelin remain to be elucidated fully, evidence now shows that ghrelin has the potential to inhibit apoptosis in rat myoblasts (Wang et al., 2018; Zhang et al., 2013, 2011). Intriguingly, optimal therapeutic time frame for ghrelin post‐MI (<24 h) appears to parallel the critical time period when cardiomyocyte apoptotic signalling is greatest.

Here, we propose that cardiac apoptotic signalling is triggered by a rapid shift in the expression of anti‐apoptotic miRs (miR‐499 and miR‐133) within hours of myocardial ischaemia, as previously reported for other cardiac‐specific miRs (Katare et al., 2011; Rawal et al., 2014a). miR‐499 is known to enhance anti‐apoptotic Bcl‐XL expression (Jia et al., 2016) and inhibit pro‐apoptotic caspase‐3 (Lew et al., 2020); hence, its anti‐apoptotic properties are well established. The link between the apoptotic properties of ghrelin and miR‐499 have not yet been explored. Likewise, miR‐133 also possesses anti‐apoptotic properties (Habibi et al., 2020), by suppressing transforming growth factor‐beta (TGF‐β) (Shan et al., 2009), and although TGF‐β is more commonly associated with profibrotic pathways (Matkovich et al., 2010), TGF‐β is also implicated in pro‐apoptotic pathways.

Given the similarity in time course between apoptotic signalling and the observed optimal therapeutic time frame of ghrelin, we hypothesize that the mechanism by which ghrelin protects the heart from apoptosis‐mediated remodelling in chronic heart failure is, at least in part, through the early modulation of anti‐apoptotic miR‐499 and miR‐133 following acute MI.

## 2 MATERIALS AND METHODS

### 2.1 Ethical Approval

All animal experimentation was approved by the Animal Ethics Committees of the University of Otago, New Zealand (D06/15). All procedures are reported in accordance with the ARRIVE guidelines and comply with *Experimental Physiology*’s policies regarding animal experiments. Experiments were conducted on male C57/B6 mice (12–14 weeks old, weighing ∼22–35 g). All mice were on a 12 h–12 h light–dark cycle at 25°C ± 1°C, group housed, and provided with food and water ad libitum. Mice were divided into eight groups, including two main categories [Acute (24 h post‐MI) and Chronic (2 weeks post‐MI)], each with four subgroups: (1) Sham+saline (*n* = 7); (2) myocardial ischaemia + saline (MI+Sal; *n* = 9); (3) Sham+ghrelin (150 µg/kg, s.c. *n* = 7); and (4) myocardial ischaemia + ghrelin (MI+Ghr; *n* = 7).

### 2.2 Myocardial ischaemia

All mice received an injection of the analgesic carprofen (5 mg/kg, s.c.) and the antibiotic Viccillin^®^ (50 mg/kg, i.m.; Meiji Seika Kaisha, Ltd, Tokyo Japan) prior to surgery. Using standard aseptic procedures, mice were anaesthetized with a 3% isoflurane in O_2_ (1 L O_2_/min) inhalation mix and maintained at 1.5%–2% of isoflurane. Mice were mechanically ventilated, and a left thoracotomy provided access to the left ventricle. A 7.0 Prolene suture was loosely placed around the left anterior descending (LAD) coronary artery, and the ligature was tied off and the LAD artery occluded in all groups of mice, except sham‐operated mice. Within 10 min of the infarct (or sham), mice received a single bolus injection (0.1 mL, s.c.) of either saline or ghrelin (150 µg/kg) (Katare et al., 2016).

Following surgery, animals were returned to their standard housing conditions, in which they remained. During this period, pain relief (carprofen) was administered once per day for 3 days, and the welfare of the mice was monitored by recording body weight, food and water consumption and by observing general appearance and behaviour.

### 2.3 Experimental protocol

#### 2.3.1 Assessment of cardiac structure and function

Echocardiography was used to evaluate indices of cardiac function immediately prior to surgical induction of MI, then again either 24 h or 2 weeks postsurgery using a Vevo 2100 System (Fujifilm VisualSonics). Mice were anaesthetized (3% isoflurane in O_2_) and body temperature thermostatically regulated. The following dimensions were measured via two‐dimensional M mode: end‐systolic volume (ESV), end‐diastolic volume (EDV), left‐ventricular anterior wall thickness at systole (LVAWS) and diastole (LVAWD), ejection fraction (EF) and fractional shortening (FS).

#### 2.3.2 Blood sampling

A 50 µL blood sample was extracted from a small tail snip at 3 h postsurgery (sham or MI) and again when mice were anaesthetized for echocardiography at 24 h post‐MI. The blood sample was centrifuged and the plasma extracted and frozen for assessment of anti‐apoptotic miR‐499 and miR‐133 (see below ‐ see section 2.3.5 below).

#### 2.3.3 Tissue collection

At the completion of each experiment (either 24 h or 2 weeks post‐MI), mice were killed by anaesthetic overdose and exsanguination, and the left ventricle was harvested, sectioned and fixed in 4% paraformaldehyde for 24 h for immunohistochemistry. Fixed samples were placed in 1× PBS, then rehydrated in 30% sucrose before storage in PBS until sectioning.

#### 2.3.4 Total RNA isolation

Total RNA was isolated from plasma samples using the miRNeasy mini kit according to the manufacturer's instructions (Qiagen), as previously described (Lew et al., 2020). The purified RNAs were quantified using a Nanodrop spectrophotometer (ThermoFisher).

#### 2.3.5 Quantitative real time‐PCR

Real‐time PCR was conformed to Minimum Information for Publication of Quantitative Real Time PCR Experiments (MIQE) guidelines (Bustin et al., 2009). Twenty nanograms of total RNA was reverse transcribed using reverse transcription primers against miR‐499 (commercial identifier, 002427–4427975) and miR‐133 (commercial identifier, 002246–4427975) (both from ThermoFisher). The complementary DNA was then amplified using Sybr green master mix (Bio‐Rad). MiR‐432‐5p (commercial identifier, 001026–4427975; ThermoFisher) was used as reference genes for plasma‐ and blood‐derived RNAs, respectively. Relative expression was expressed as 2^−Δ^ *^Ct^* (Lew et al., 2020).

#### 2.3.6 In situ detection of apoptotic cardiomyocytes

The paraformaldehyde‐fixed ventricular tissue samples were coated in OCT and cut into 7‐µm‐thick sections. Cardiomyocyte apoptosis at the border zone of the infarct was detected using terminal deoxynucleotidyl transferase (TdT)‐mediated dUTP‐biotin nick end‐labelling (TUNEL) staining according to the manufacturer's protocol (Invitrogen). Total nuclei at the border zone of the infarct was determined using DAPI co‐stain. An investigator blinded to the identity and treatment of the mice captured several images for each slide. Then, for each image, the investigator counted the total number of cells and TUNEL‐positive cells within three evenly distributed segments, 200 µm × 200 µm, across the border zone, and the average of all segments was representative of total infarct border zone. All slides were visualized under a ×20 dry objective lens using an Olympus BX51 fluorescence microscope. Data are expressed as the proportion (percentage) of TUNEL‐positive‐stained cells to all DAPI‐stained cardiomyocytes.

#### 2.3.7 Measurement of infarct size (2 weeks post‐MI only)

The left ventricle was sectioned horizontally through the middle of the infarct area. Sections of the infarcted area, 5 µm thick, were stained with Masson's Trichrome stain, mounted for light microscopy examination and photographed. Infarct size was determined by measuring the entire endocardial area, then measuring the segment of the endocardial area that composed the infarcted portion (Schwenke et al., 2012). Images were analysed using ImageJ software (https://imagej.net/ij/), and infarct size was presented as a percentage of the left free ventricular wall.

**FIGURE 1:**
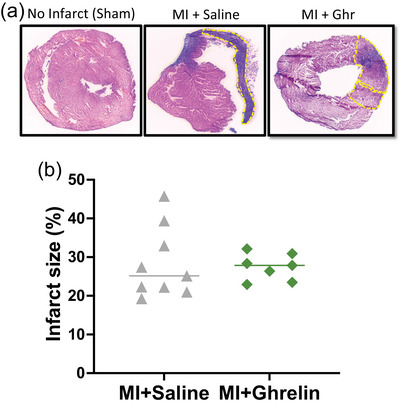
(a) Representative images of Masson's Trichrome stained cardiac slices in a normal, infarcted heart treated with either saline or ghrelin, showing clear demarcation of the infarct region (yellow border). (b) Quantitative scatter plot showing infarct size (2 weeks post‐MI) in MI mice treated with saline (*n* = 9) or 150 µg/kg ghrelin (*n* = 7). There was no significant difference (Student's unpaired *t*‐test) in infarct size between mice treated with saline (19.3%–45.8%) vs. ghrelin (22.9%–32.1%). Abbreviations: Ghr, ghrelin; MI, myocardial infarction.

Importantly, in this study the size of the infarct of the left ventricular wall did not differ significantly between MI+Saline and MI+Ghrelin mice (Figure 1).

### 2.4 Statistical analysis

All statistical analyses were performed using GraphPad Prism Software (v.10.0). Data were expressed as the mean ± SD. A two‐way ANOVA was used to test significance for: (1) differences between sham and MI at acute and chronic stages post‐MI; and (2) the effect of ghrelin vs. saline (placebo) on individual variables for sham and MI groups. Repeated‐measures correction was used to test for significant changes in echocardiography measurements pre‐ vs. post‐MI. Where significance was reached, Tukey's multiple comparisons test was performed to correct for multiple comparisons. A *p*‐value of <0.05 was predetermined as the level of significance for all statistical analyses.

## 3 RESULTS

### 3.1 Effect of myocardial ischaemia on circulating anti‐apoptotic microRNAs and myocyte apoptosis

**FIGURE 2:**
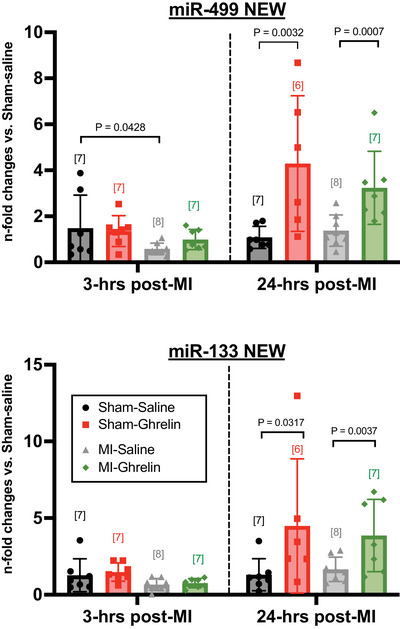
Quantitative scatter plot bar graphs (mean ± SD) showing the relative levels of circulating anti‐apoptotic miR‐499 (a) and anti‐apoptotic miR‐133 (b) in mice measured 3 or 24 h post‐myocardial infarction (MI) or sham surgery (Sham). MI and Sham mice were treated with saline [Sham+saline (*n* = 7 for both miR‐499 and miR‐133); MI+saline (*n* = 8 for miR‐499 and miR‐133)] or 150 µg/kg ghrelin [Sham+ghrelin (*n* = 7 for miR‐499 and *n* = 6 for miR‐133); MI+ghrelin (*n* = 7 for miR‐499 and miR‐133)]. The *p*‐values denote significant differences between Sham vs. MI; or saline vs. ghrelin (two‐way ANOVA, with Tukey's *post‐hoc* test to correct for multiple comparisons). Relative expression of miR was normalized to miR‐432 and expressed as 2^−Δ^ *^Ct^*. The population size for each group is indicated in the square brackets.

**FIGURE 3:**
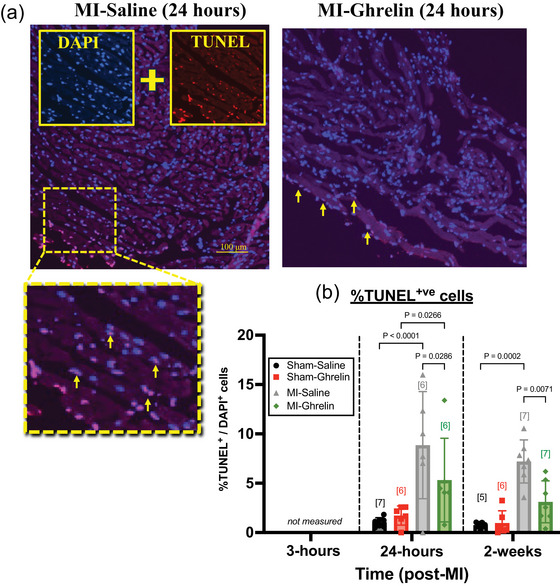
(a) Representative image showing positive staining of terminal deoxynucleotidyl transferase‐mediated dUTP‐biotin nick end‐labelling (TUNEL)‐stained nuclei (×20 objective lens) 24 h post‐MI, which represents cardiomyocyte apoptosis, in cardiac slices taken from myocardial infarction (MI) mice treated with saline (MI‐Saline) or ghrelin (MI‐Ghrelin). Inset: zoomed‐in image showing examples of individual TUNEL‐positive cardiomyocytes (yellow arrows). (b) Quantitative scatter plot bar (mean ± SD) showing the number of TUNEL‐positive cardiomyocytes (apoptosis) as a proportion of overall cell count (DAPI) in mice measured 24 h or 2 weeks post‐myocardial infarction (MI) or sham surgery. MI and Sham mice were treated with saline [Sham+saline (*n* = 7 for 24 h postsurgery and *n* = 5 for 2 weeks postsurgery); MI+saline (*n* = 6 for 24 h post‐MI and *n* = 7 for 2 weeks post‐MI)] or 150 µg/kg ghrelin [Sham+ghrelin (*n* = 6 for both 24 h and 2 weeks postsurgery); MI+ghrelin (*n* = 6 for 24 h post‐MI and *n* = 7 for 2 weeks post‐MI)]. The *p*‐values denote significant differences between Sham vs. MI; or saline vs. ghrelin (two‐way ANOVA, with Tukey's *post‐hoc* test to correct for multiple comparisons). The population size for each group is indicated in the square brackets.

Within 3 h of an acute MI, the level of anti‐apoptotic miR‐499 appeared to be 61% lower compared with sham (control) mice (*p* = 0.0428; *n* = 7), although by 24 h post‐MI, the level of miR‐499 was similar to that of sham mice (Figure 2a). miR‐133 remained unaltered at 3 and 24 h post‐MI (Figure 2b). Although we did not confirm cardiomyocyte apoptosis at 3 h post‐MI, severe apoptosis was evident at 24 h post‐MI by a significant 796% increase in the proportion of TUNEL‐positive cardiomyocytes (*p* < 0.0001; *n* = 6; Figure 3). Cardiomyocyte apoptosis continued to be significantly elevated at 2 weeks post‐MI, although no more so than that observed at 24 h post‐MI.

### 3.2 Ghrelin increases the levels of anti‐apoptotic microRNAs to ameliorate apoptosis

The administration of ghrelin following acute MI prevented the significant decrease in the levels of miR‐499 at 3 h post‐MI, a decrease that was evident in saline‐treated MI mice, but did not impact on the levels of miR‐133 (Figure 2). Most notably, by 24 h postsurgery the levels of both miR‐499 and miR‐133 were significantly elevated in all ghrelin‐treated mice, both sham (*p* = 0.0032 and *p* = 0.0317, respectively; *n* = 7) and MI mice (*p* = 0.0007 and *p* = 0.0037, respectively; *n* = 7; Figure 2). For MI mice, the ghrelin‐mediated increase in anti‐apoptotic miR‐499 and miR‐133 appeared to ameliorate the severity of cardiomyocyte apoptosis by 24 h post‐MI, although not significantly; the effect achieved significance by 2 weeks post‐MI (*p* = 0.0071; *n* = 7), compared with the respective saline‐treated MI mice (Figure 3).

### 3.3 Cardiac dysfunction and remodelling following MI is attenuated by ghrelin

**FIGURE 4:**
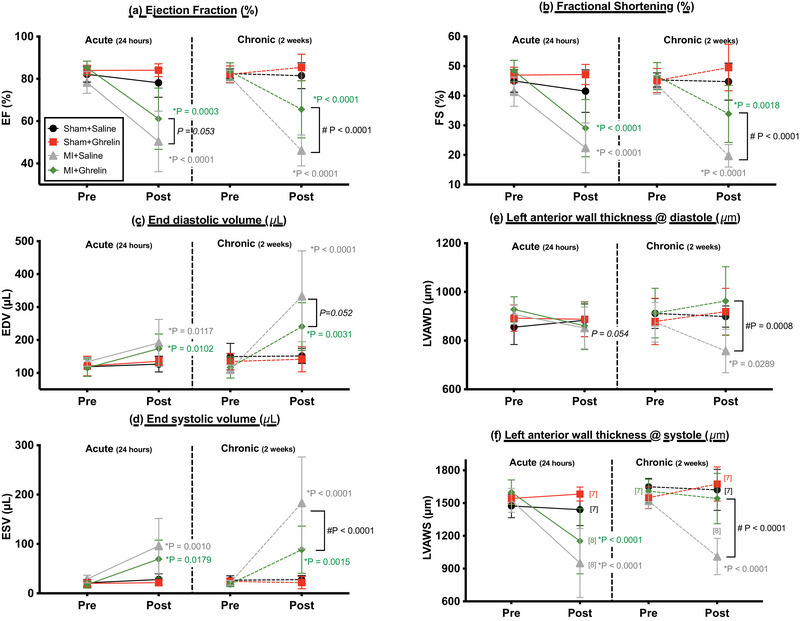
Line graphs showing changes in cardiac function and structure in mice before (Pre) and after (Post) acute myocardial infarction (or sham surgery). Post‐MI measurements were recorded either 24 h post‐MI (Acute) or 2 weeks post‐MI (Chronic). Measures were made for: (a) ejection fraction (EF); (b) fractional shortening (FS); (c) end‐diastolic volume (EDV); (d) end‐systolic volume (ESV); (e) left ventricular anterior wall thickness at end systole (LVAWS); and (f) left ventricular anterior wall thickness at end diastole (LVAWD). The MI and Sham mice were treated with saline (Sham+saline; MI+saline) or 150 µg/kg ghrelin (Sham+ghrelin; MI+ghrelin). The population size for each group is indicated in the square brackets (see panel f). Data are presented as the mean ± SD. ^*^ *p*‐values for comparing pre‐ vs. post‐MI. ^#^ *p*‐values for comparing MI‐Saline vs. MI‐Ghrelin (two‐way ANOVA, with Tukey's *post‐hoc* test to correct for multiple comparisons). All individualised data are available in the supplementary material.

Acute MI was associated with cardiac dysfunction and ventricular remodelling in saline‐treated MI mice by 24 h post‐MI, which was exacerbated further by 2 weeks post‐MI (Figure 4). This was evident by ventricular dilatation [e.g., 199% increase in EDV by 2 weeks (*p* < 0.0001; *n* = 8); Figure 4c] and remodelling [e.g., 33% and 14% decrease in LVAWS (*p* < 0.0001) and LVAWD (*p* = 0.0289), respectively (*n* = 8)], impaired contractility that was evident by a 771% increase in ESV (*p* < 0.0001; *n* = 8; Figure 4d) and, ultimately, an impaired ejection fraction (decrease from 80% to 46%; *p* < 0.0001, *n* = 8; Figure 4a). Yet, in those mice that received a single bolus of ghrelin following the acute MI (i.e., MI+Ghrelin), indices of cardiac function (EDV, ESV and EF) were significantly improved compared with saline‐treated MI mice (Figure 4a, c, d), and ventricular remodelling was significantly attenuated (i.e., LVAWS and LVAWD were unchanged; Figure 4e, f).

## 4 DISCUSSION

The cardioprotective properties of ghrelin have become well cemented within the scientific and clinical literature. The underlying mechanisms by which ghrelin act throughout the cardiovascular system have remained an ongoing area of intense research for over two decades. This study further advances the dogma that ghrelin improves cardiac function and structure after an MI, by demonstrating the ability of ghrelin to ameliorate ischaemia‐induced cardiomyocyte apoptosis. Importantly, this study also provides new evidence implicating miR‐499 and miR‐133 as likely mechanistic targets underpinning the anti‐apoptotic properties of ghrelin.

One of the most detrimental consequences of myocardial ischaemia is the irreversible structural remodelling of the heart that subsequently reduces the optimal functional capacity. This structural deformation of the heart is driven, at least in part, by overactivation of cardiomyocyte apoptotic signalling, a process that is otherwise meticulously controlled (Farokhian et al., 2023). Currently, there is no effective therapy to mitigate the ischaemic‐induced cardiomyocyte apoptosis, because early detection of enhanced apoptosis remains elusive.

Although there are a plethora of reports describing the cardioprotective properties of ghrelin in ischaemic heart disease, accumulating evidence in the literature is now establishing ghrelin as a potent anti‐apoptotic peptide (Katare et al., 2016; Neale et al., 2020; Wang et al., 2018; Zhang et al., 2013, 2011).

In agreement, the data in the present study also demonstrate the ability of ghrelin to impede ischaemia‐induced apoptotic signalling. As a result, this anti‐apoptotic potential of ghrelin is likely to underpin the reduced structural remodelling and thus, improved cardiac function following MI, as observed in the present study and as previously described (Schwenke et al., 2012).

Although this study does not provide the definitive mechanistic data on how ghrelin suppresses apoptosis, we do provide new observational evidence suggesting that ghrelin targets specific anti‐apoptotic miR‐499 and miR‐133. Ghrelin appeared to increase miR‐499 and miR‐133 levels irrespective of myocardial ischaemia, although the beneficial downstream effect on myocyte apoptosis was apparent only in the event of myocardial ischaemia, that is, the ghrelin‐induced increase in miR‐499 and miR‐133 had no impact on apoptotic signalling in sham‐operated animals.

Ghrelin has previously been shown to modulate various microRNAs associated with ischaemia‐related angiogenesis and inflammation (Katare et al., 2016; Neale et al., 2020), hypoxia‐inducible factor (Mirzaei Bavil et al., 2022) and angiotensin‐mediated myocyte apoptosis (Wang et al., 2018). Moreover, Neale et al. (2020) observed that, in ghrelin‐knockout mice, ischaemia‐induced apoptosis was exacerbated, and the expression of pro‐angiogenic miR‐126 was adversely reduced.

The downstream targets by which miR‐499 elicits anti‐apoptotic properties were not elucidated in the present study. However, Wang et al. (2014) demonstrated that inhibition of miR‐499 in H_2_O_2_‐treated myoblasts increased apoptosis via upregulation of pro‐apoptotic cytochrome *c* and caspase‐3. Moreover, miR‐499 overexpression abolished apoptosis by silencing pro‐apoptotic genes that are direct targets of miR‐499 (e.g., *Pdcd4*, *Pacs2* and *Dyrk2*), all of which are upstream mediators of apoptotic signalling that ultimately activates pro‐apoptotic caspase‐3. In agreement, Lew et al. (2020) reported that cardiomyocyte apoptosis in diabetes was closely linked with downregulation of miR‐499 owing to an increase in caspase‐3. They also reported that upregulation of miR‐499 inhibited caspase‐3 and, thus, prevented the diabetes‐mediated cardiac apoptosis and improved cardiac function.

Despite the compelling and new observational results of this study, one limitation is that the lack of mechanistic data means that a definitive link between the anti‐apoptotic properties of ghrelin and the modulation of miR‐499 and miR‐133 remains speculative. However, considering the evidence in the literature that miRs are potent modulators of pro‐apoptotic genes, it is an attractive notion that stabilizing the apoptotic pathway in myocardial ischaemia, through the specific modulation of miRs (including miR‐499 and miR‐133), becomes a transformative strategy for clinical treatment of heart failure.

## 5 CONCLUSION

In conclusion, the mechanisms that underpin the cardioprotective properties of ghrelin are multifaceted. Here, we have used an experimental animal model to demonstrate that ghrelin ameliorates cardiomyocyte apoptosis following acute myocardial infarction to mitigate cardiac structural remodelling and dysfunction effectively. Importantly, the data also provide new evidence suggesting that this anti‐apoptotic property of ghrelin might be mediated through modulation of miR‐499 and miR‐133, although further mechanistic studies are required to verify these observations.

## AUTHOR CONTRIBUTIONS

All authors were responsible for designing and completing the overall study. Moreover, Rajesh Katare oversaw and completed all PCR experimental work and analysis, Jeffrey R. Erickson completed all TUNEL experimental assessment of cardiomyocyte apoptosis, and Daryl O. Schwenke completed all cardiac structural and functional experiments. Daryl O. Schwenke was responsible for drafting the initial manuscript, and both Rajesh Katare and Jeffrey R. Erickson provided edited versions of the final manuscript. All authors read and approved the final version of the manuscript and agree to be accountable for all aspects of the work in ensuring that questions related to the accuracy or integrity of any part of the work are appropriately investigated and resolved. All persons designated as authors qualify for authorship, and all those who qualify for authorship are listed.

## CONFLICT OF INTEREST

The authors declare no conflict of interest.

## Supporting information




**Supplementary Table 1**. Individual data points from Figure 4 showing the changes in cardiac function and structure in mice before (Pre) and after (Post) acute myocardial infarction (or sham). Post‐MI measurements were recorded either 24 hours post‐MI (Acute) or 2 weeks post‐MI (Chronic).

## Data Availability

Some or all datasets generated during and/or analysed during the present study are not publicly available but are available from the corresponding author on reasonable request.
